# Supplementary material for: Combining citizen science, phylogenetics, and bioacoustics to inform taxonomy and conservation of the Near Threatened Proceratophrys paviotii (Anura, Odontophrynidae)
Source: PeerJ. 2024 Oct 23;12:e17990. doi: 10.7717/peerj.17990 (PMC11512549; doi:10.7717/peerj.17990)
Supplement: Supplemental Information 1 [file peerj-12-17990-s001.docx]

| **Species** | **Genbank Acession** | **Reference** |
| --- | --- | --- |
| *Cycloramphus boraceiensis* | DQ283097 | Frost et al. (2006) |
| *Macrogenioglottus alipioi* | FJ685684 | Amaro et al. (2009) |
| *Macrogenioglottus alipioi* | FJ685685 | Amaro et al. (2009) |
| *Odontophrynus* | JX564880 | Zhang et al. (2013) |
| *Odontophrynus achalensis* | KP295642 | Faivovich et al (2014) |
| *Odontophrynus toledoi* | FJ685686 | Amaro et al. (2009) |
| *Odontophrynus carvalhoi* | FJ685687 | Amaro et al. (2009) |
| *Odontophrynus cultripes* | FJ685688 | Amaro et al. (2009) |
| *Odontpphrynus asper* | AY843704 | Faivovich et al. (2005) |
| *Proceratophrys* aff. *ararype* | FJ685694 | Amaro et al. (2009) |
| *Proceratophrys appendiculata* | KF214151 | Dias et al. (2013) |
| *Proceratophrys appendiculata* | KF214152 | Dias et al. (2013) |
| *Proceratophrys ararype* | KX858852 | Mângia et al. (2018) |
| *Proceratophrys ararype* | KX858853 | Mângia et al. (2018) |
| *Proceratophrys ararype* | KX858854 | Mângia et al. (2018) |
| *Proceratophrys avelinoi* | DQ283039 | Frost et al. (2006) |
| *Proceratophrys avelinoi* | FJ685691 | Amaro et al. (2009) |
| *Proceratophrys avelinoi* | KP295643 | Faivovich et al (2014) |
| *Proceratophrys belzebul* | KF214154 | Dias et al. (2013) |
| *Proceratophrys belzebul* | KF214155 | Dias et al. (2013) |
| *Proceratophrys belzebul* | KF214156 | Dias et al. (2013) |
| *Proceratophrys bigibbosa* | FJ685692 | Amaro et al. (2009) |
| *Proceratophrys bigibbosa* | MG798659 | Dias et al. (2018) |
| *Proceratophrys bigibbosa* | MG798660 | Dias et al. (2018) |
| *Proceratophrys boiei* N1 | JN814630 | Amaro et al. (2012) |
| *Proceratophrys boiei* N1 | JN814653 | Amaro et al. (2012) |
| *Proceratophrys boiei* N1 | JN814662 | Amaro et al. (2012) |
| *Proceratophrys boiei* N2 | JN814592 | Amaro et al. (2012) |
| *Proceratophrys boiei* N2 | JN814620 | Amaro et al. (2012) |
| *Proceratophrys boiei* N2 | JN814648 | Amaro et al. (2012) |
| *Proceratophrys boiei* S | JN814586 | Amaro et al. (2012) |
| *Proceratophrys boiei* S | JN814612 | Amaro et al. (2012) |
| *Proceratophrys boiei* S | JN814660 | Amaro et al. (2012) |
| *Proceratophrys brauni* | KU495472 | Lyra et al. (2016) |
| *Proceratophrys concavitympanum* | KX858855 | Mângia et al. (2018) |
| *Proceratophrys cristiceps* | FJ685695 | Amaro et al. (2009) |
| *Proceratophrys cristiceps* | MF953400 | Mângia et al. (2018) |
| *Proceratophrys cristiceps* | MF953401 | Mângia et al. (2018) |
| *Proceratophrys cururu* | FJ685696 | Amaro et al. (2009) |
| *Proceratophrys cururu* | KU495477 | Lyra et al. (2016) |
| *Proceratophrys cururu* | KU495478 | Lyra et al. (2016) |
| *Proceratophrys goyana* | FJ685697 | Amaro et al. (2009) |
| *Proceratophrys goyana* | KU495479 | Lyra et al. (2016) |
| *Proceratophrys itamari* | FJ685699 | Amaro et al. (2009) |
| *Proceratophrys itamari* | KF214142 | Dias et al. (2013) |
| *Proceratophrys itamari* | KF214147 | Dias et al. (2013) |
| *Proceratophrys izecksohni* | KF214157 | Dias et al. (2013) |
| *Proceratophrys izecksohni* | KU495483 | Lyra et al. (2016) |
| *Proceratophrys kaingang* | MW916089 | Santana et al. (2021a) |
| *Proceratophrys kaingang* | MW916090 | Santana et al. (2021a) |
| *Proceratophrys kaingang* | MW916088 | Santana et al. (2021a) |
| *Proceratophrys korekore* | MW889930 | Santana et al. (2021b) |
| *Proceratophrys korekore* | MW889928 | Santana et al. (2021b) |
| *Proceratophrys korekore* | MW889929 | Santana et al. (2021b) |
| *Proceratophrys laticeps* | FJ685698 | Amaro et al. (2009) |
| *Proceratophrys mantiqueira* | KF214143 | Dias et al. (2013) |
| *Proceratophrys melanopogon* | KF214140 | Dias et al. (2013) |
| *Proceratophrys melanopogon* | KF214149 | Dias et al. (2013) |
| *Proceratophrys minuta* | JX982965 | Teixeira Jr et al (2012) |
| *Proceratophrys minuta* | JX982966 | Teixeira Jr et al (2012) |
| *Proceratophrys moratoi* | FJ685689 | Amaro et al. (2009) |
| *Proceratophrys moratoi* | MT196403 | Magalhães et al. (2020) |
| ***Proceratophrys paviotii*** | **PP442191** | **Present work** |
| ***Proceratophrys paviotii*** | **PP442192** | **Present work** |
| ***Proceratophrys paviotii*** | **PP442193** | **Present work** |
| *Proceratophrys pombali* | KF214144 | Dias et al. (2013) |
| *Proceratophrys pombali* | KF214148 | Dias et al. (2013) |
| *Proceratophrys redacta* | JX982967 | Teixeira Jr et al (2012) |
| *Proceratophrys redacta* | JX982968 | Teixeira Jr et al (2012) |
| *Proceratophrys renalis* | FJ685700 | Amaro et al. (2009) |
| *Proceratophrys renalis* | JN814584 | Amaro et al. (2012) |
| *Proceratophrys salvatori* | MT196397 | Magalhães et al. (2020) |
| *Proceratophrys salvatori* | MT196399 | Magalhães et al. (2020) |
| *Proceratophrys schirchi* | FJ685701 | Amaro et al. (2009) |
| *Proceratophrys strussmannae* | KU495473 | Lyra et al. (2016) |
| *Proceratophrys strussmannae* | LTJV180 | Santana et al. (2021b) |
| *Proceratophrys strussmannae* | MHO117 | Santana et al. (2021b) |
| *Proceratophrys strussmannae* | MHO290 | Santana et al. (2021b) |
| *Proceratophrys strussmannae* | MHO309 | Santana et al. (2021b) |
| *Proceratophrys tupinamba* | KF214158 | Dias et al. (2013) |
| *Proceratophrys tupinamba* | KF214159 | Dias et al. (2013) |
| *Proceratophrys tupinamba* | KF214160 | Dias et al. (2013) |
| *Proceratophrys velhochico* | MT537176 | Mângia et al. (2022) |
| *Proceratophrys velhochico* | MT537177 | Mângia et al. (2022) |
| *Thoropa miliaris* | FJ685682 | Amaro et al. (2009) |
